# Combining voltammetric and mass spectrometric data to evaluate iron organic speciation in subsurface coastal seawater samples of the Ross sea (Antarctica)

**DOI:** 10.1007/s11356-022-23975-w

**Published:** 2022-11-12

**Authors:** Davide Vivado, Francisco Ardini, Annalisa Salis, Gianluca Damonte, Paola Rivaro

**Affiliations:** 1 Department of Chemistry and Industrial Chemistry, University of Genova, Via Dodecaneso 31, 16146 Genoa, Italy; 2 Department of Experimental Medicine, Section of Biochemistry, University of Genova, Viale Benedetto XV 1, 16132 Genoa, Italy

**Keywords:** Iron, Speciation, Sea water, CLE-AdSV, HPLS-ESI–MS/MS, Ross sea

## Abstract

Iron (Fe) is the most important trace element in the ocean, as it is required by phytoplankton for photosynthesis and nitrate assimilation. Iron speciation is important to better understand the biogeochemical cycle and availability of this micronutrient, in particular in the Southern Ocean. Dissolved Fe (dFe) concentration and speciation were determined in 24 coastal subsurface seawater samples collected in the western Ross sea (Antarctica) during the austral summer 2017 as part of the CELEBeR (CDW Effects on glacial mElting and on Bulk of Fe in the Western Ross sea) project. ICP-DRC-MS was used for dFe determination, whereas CLE-AdSV was used to obtain the concentration of complexed and free dFe, of the ligands, and the values of the stability constants of the complexes. Dissolved Fe values ranged from 0.4 to 2.5 nM and conditional stability constant (log*K*’_**Fe’L**_) from 13.0 to 15.0, highlighting the presence of Fe-binding organic complexes of different stabilities. Principal component analysis (PCA) allowed us to point out that Terra Nova Bay and the neighboring area of Aviator and Mariner Glaciers were different in terms of chemical, physical, and biological parameters. A qualitative investigation on the nature of the organic ligands was carried out by HPLC–ESI–MS/MS. Results showed that siderophores represented a heterogeneous class of organic ligands pool.

**Supplementary Information:**

The online version contains supplementary material available at 10.1007/s11356-022-23975-w.

## Introduction

Iron (Fe) is the most important trace element in the ocean ecosystem, being a micronutrient required for phytoplankton growth, and hence involved in marine primary productivity and carbon export (Ibisanmi et al. 2011). Given its role in primary production, Fe can regulate atmospheric carbon dioxide (CO_2_) concentration and indirectly the global climate system. The oceanic concentration of Fe is low (typically < 1 nM in deep waters) which is caused by its poor solubility and biological uptake (Liu and Millero 2002; Abualhaija and van den Berg 2014). Dissolved Fe concentration is very low in most of the Southern Ocean, with values as low as 50 pM (De Baar et al. 1999), and these regions are called high nutrient low chlorophyll (HNLC). In particular, these areas are characterized by high concentrations of macronutrients, but low amounts of phytoplankton biomass, measured in terms of chlorophyll (Chl) concentration (Gledhill and Buck 2012). This restriction in phytoplankton growth seems to be the result of Fe limitation, in accordance with Martin’s iron hypothesis (Martin 1990; Worsfold et al. 2014), according to which the deficiency of this element is the factor responsible for the existence of HNLC areas. Some areas of the Southern Ocean have recently been defined as “green areas” and “blue desert areas,” based on the average concentrations of chlorophyll-*a* (Chl-*a*) measured in the summer season. Green areas (West Pacific, West Atlantic, and West Indian) are characterized by a concentration of Chl-*a* up to 5 mg m^−3^; on the contrary in blue desert areas (East Pacific, West Atlantic, and East Indian), the Chl-*a* concentration is less than 0.1 mg m^−3^. The presence of green and blue desert areas has been linked to the melting rates of sea ice and the consequent release of Fe in surface waters. In green areas, the melting rate of ice is greater, so macro- and micronutrients are released into the water column, allowing phytoplankton growth (Meguro et al. 2004).

Almost all dissolved iron (dFe) in seawater is bound to organic ligands (*L*) of largely unknown identity (Gledhill and Buck 2012; Buck et al. 2015). These ligands increase the solubility of Fe and without them the concentration of dFe is thought to be significantly lower, due to the “scavenging” phenomena and for the formation and precipitation of Fe oxides and hydroxides (Ibisanmi et al. 2011).

Competitive ligand equilibration–adsorptive stripping voltammetry (CLE-AdSV) is the most common technique to measure the concentration of complexed and free dFe together with the concentration and binding strength of *L* (Croot et al. 2004; Gerringa et al. 2008; Laglera and Monticelli 2017). On the basis of CLE-AdSV results, *L* are generally referred to as either strong (*L*_1_ type) or weak (*L*_2_ type) ligands, though several additional ligand classes have also been reported (Hunter and Boyd 2007; Gledhill and Buck 2012; Bundy et al. 2018). However, through CLE-AdSV molecular composition of ligands cannot be inferred.

On the contrary, high-performance liquid chromatography–electrospray ionization–tandem mass spectrometry (HPLC–ESI–MS/MS) provides a new powerful approach to identifying the unknown ligands involved in dFe speciation. This technique allows the separation of the analytes through capabilities of HPLC, and it provides structural characterization by MS following the fragmentation pattern in the MS/MS spectra (McCormack et al. 2003).

Iron biogeochemistry in the Ross Sea has been investigated in some recent studies, with particular attention to dFe (Gerringa et al. 2015a; McGillicuddy et al. 2015; Rivaro et al. 2019). Since the Ross Sea is a continental shelf zone, dFe inputs are higher than in the open Southern Ocean. In addition to vertical mixing and atmospheric inputs, there are continental and sediment inputs, intrusion of waters of circumpolar origin (Circumpolar Deep Water, CDW) and release of material from continental glacial platforms (ice shelf) and icebergs (Gerringa et al. 2015b; Henley et al. 2020). For these reasons, primary production in the Ross Sea is estimated to be about 179 g C m^− 2^ year^−1^, which is the highest of the coastal regions of the Southern Ocean (Arrigo et al. 2008; Smith et al. 2014). Despite this, the release rates of Fe during the spring/summer season can be limited, affecting primary production and consequently the entire phytoplankton community.

The different phytoplankton blooms in the Ross sea occur in different seasons and areas. Primesiophytes dominate in springtime in the open polynyas of the central-southern region, whereas diatoms dominate in summer in the western and eastern portion of the Ross sea. The temporal and spatial distribution of these groups has been related to the concentration of dFe and the availability of light, in turn linked to the presence of ice coverage and vertical mixing (Smith et al. 2014; Henley et al. 2020).

Basal melting of glaciers (e.g., Nansen, Mariner, and Aviator) provides fresh water to the western coastal area the Ross sea (Rignot et al. 2013). Glaciers could thus largely contribute to the dFe pool, potentially stimulating the biological pump and therefore to the transfer of CO_2_.

The CELEBeR (CDW Effects on glacial mElting and on bulk of Fe in the Western Ross sea) project aimed at constraining the sources, stocks, and flows of Fe in the western Ross sea ecosystem. In particular, the specific objectives were to elucidate how the dFe chemical speciation controls and is controlled by phytoplankton and bacterioplankton communities and how the different sources impact Fe speciation and bioavailability for polar microorganisms.

Here, we present the distribution of dFe and its chemical speciation in the subsurface waters sampled in a well-studied system such as Terra Nova Bay (TNB) polynya and in the neighboring area of Aviator and Mariner Glaciers (AMG), which to date has not been studied in a systematic way. The data will be discussed using a multivariate approach that will help outlining the correlations among dFe concentration, dFe speciation parameters, and biogeochemical patterns. In order to better investigate the Fe chemical speciation and cycling, we coupled our CLE-AdSV data with HPLC–ESI–MS/MS analyses.

To our knowledge, this work is the first study that compares voltammetric and mass spectrometric measurements of Fe-binding ligands in the western Ross sea.

## Materials and methods

### Sample collection and processing

**Fig. 1:**
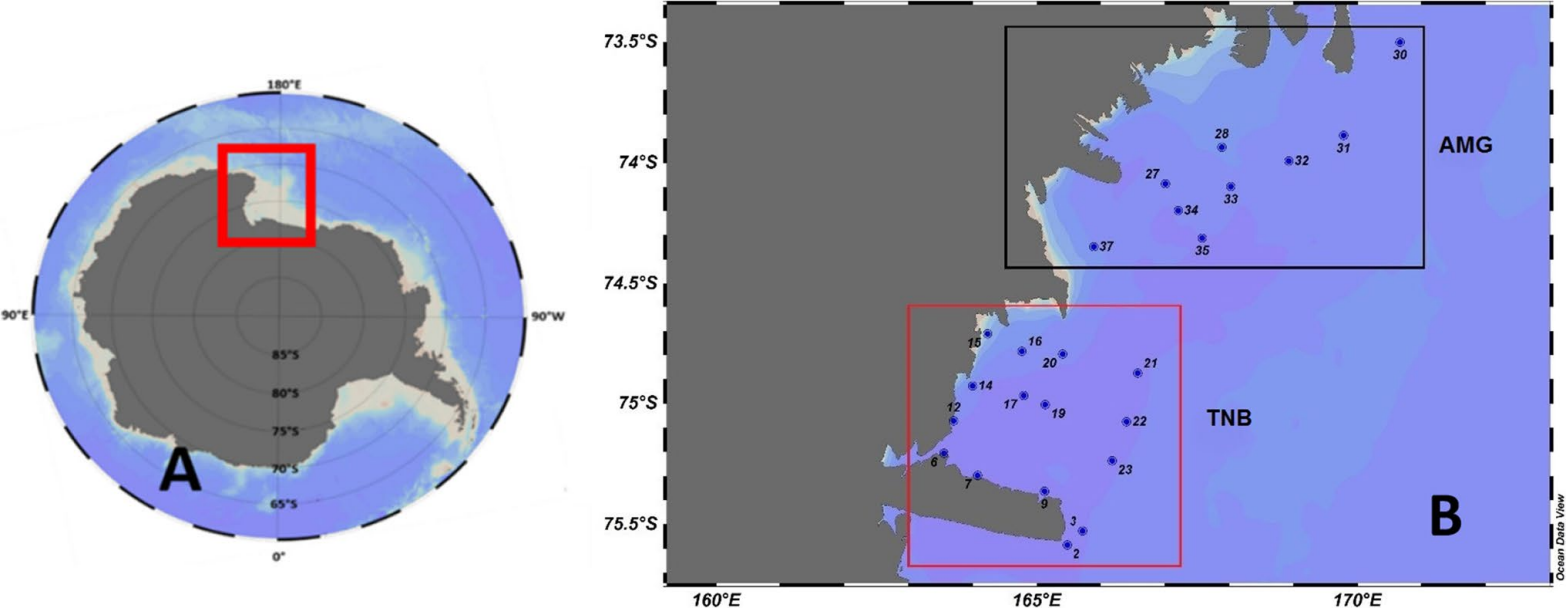
**A** Position of the Ross Sea in the Antarctic continent. **B** Sampling stations of the CELEBeR project for the areas of Terra Nova Bay (TNB) and the Aviator and Mariner Glaciers (AMG)

Samples were collected onboard the R.V. Italica from the ninth to the twenty-first of January 2017 in two different coastal sub-areas of the western Ross sea: Terra Nova Bay (TNB) and Aviator and Marine Glaciers (AMG) area (Fig. 1, Table S1) as part of the Italian National Program of Research in Antarctica (PNRA) activities.

A Sea Bird Electronics SBE9/11plus CTD profiler with two pairs of temperature-conductivity sensors was employed to acquire conductivity, temperature, and depth data. The CTD was coupled to a SBE 23 dissolved oxygen (O_2_) sensor and to a Chelsea Aquatrack III fluorometer for measuring the oxygen concentration and the fluorescence, respectively. The samples were collected at subsurface (depth 10–40 m) where the fluorescence maxima were observed.

A 5-L teflon-lined GO-FLO bottle (General Oceanics Inc.) was used to collect seawater samples for Fe analysis. The bottle was deployed on a Kevlar 6-mm diameter line, and it was sealed using a polyvinyl chloride (PVC) messenger. After collecting the sample, the bottle was covered with plastic bags to reduce contamination.

Two liters of the water samples were collected in polyethylene bottles and immediately filtered using 0.45-µm pore-sized polycarbonate (PC) filters previously washed in diluted suprapur hydrochloric acid (HCl) (Merck, Darmstadt, Germany) using a clean conditions filtration system, limiting filtration time to 1–2 h. This custom built filtration apparatus was successfully tested for trace metal analysis of Antarctic water samples (Rivaro et al. 2011). Aliquots of 200 mL were collected and frozen at − 20 °C. Suprapur® 65% nitric acid (HNO_3_) (Merck, Darmstadt, Germany) was used for the cleaning of materials.

A SBE 32 plastic coated carousel sampler was used to collect water samples from 24 12-L Niskin bottles for O_2_, nutrients, and carbonate system parameters. Seawater samples for carbonate analyses were collected at selected depths and were poured into 500-mL borosilicate glass bottles following standard operating procedures (Dickson et al. 2007). The samples were poisoned in the container with saturated HgCl_2_ to stop biological activity. Samples were then stored in dark, cold (+ 4 °C) conditions. Sub-samples for the determination of nutrients (silicate, phosphate, nitrate plus nitrite) were collected directly from the Niskin bottle, filtered through a 0.7-mm GFF filter and stored at − 30 °C in 50-mL low-density polyethylene containers, prior to analysis.

### Total dissolved iron analysis

Ultrapure water from a Milli-Q system (Millipore, Watford, Hertfordshire, UK) was used throughout. Trace Select® Ultra 65% HNO_3_ from Sigma–Aldrich (St. Louis, MO, USA) was used for the final stage of the cleaning procedure of materials and for the preparation of standards and samples.

Under a laminar flow hood, 50.0 g of acidified seawater sample (pH 1.8) and 500 μL of concentrated NH_4_OH (Trace Select® Ultra Sigma–Aldrich) were added into an acid-cleaned 50-mL centrifuge tube; after 1.5 min, it was shaken and left to stand for 3 min. The sample was centrifuged at 3000 rpm for 3 min; the supernatant was discarded and the pellet was re-dissolved in 5 mL of 1% (v/v) HNO_3_.

Inductively coupled plasma mass spectrometer (ICP-DRC-MS Perkin Elmer-Sciex Elan DRC II, Concord, Ontario, Canada) equipped with a PFA-ST microconcentric pneumatic nebulizer sample introduction system (Elemental Scientific, Omaha, NE, USA), operating with a 20-mL inner volume Cinnabar spray chamber (Glass Expansion, Melbourne, Australia) was used for dFe determination. Full details of the procedure and of the instrumentation used by our research group are given in Ardini et al. (2011). The detection limit (LOD) of the method was computed as three times the standard deviation of 13 blanks. The LOD resulted 0.09 nM, which is adequate for our analytical purposes. Accuracy (trueness and precision) was verified by the analysis of the Geotraces GS seawater reference material. Accurate results were obtained for the dFe determination (found concentration 0.500 ± 0.030 nM and certified value 0.546 ± 0.046 nM) with an error of 8.42%. Precision was satisfactory with RSD% of 5.86%.

Since the seawater samples have similar composition, calibration was performed by the addition calibration technique, a simplification of the standard addition method, in which the slope obtained for a single representative sample is used for the calibration of the other samples (Ardini et al. 2011; Wu and Boyle 1998).

### Iron organic speciation analysis by CLE-AdSV

Under a laminar flow work area at ambient temperature, 250 μL of 0.1 mM methanolic solution of 2,3-dihydroxynaphtalene (DHN) (Sigma–Aldrich, Saint Louis, Missouri, USA) were added to 50 mL homogenized sample. Aliquots of 7 mL of the sample/ligand solution were pipetted in 7 pre-cleaned 15-mL tubes with incremental additions of Fe(III) standard solution, with approximately four increments in the competition region and three increments where ligands are saturated. Samples were left to equilibrate overnight (ca. 15 h) in the dark to prevent the slow oxidation of DHN. Before the analysis of each aliquot, 300 μL of 0.4 M potassium bromate/0.1 M HEPPS (4-(2-hydroxyethyl)-piperazine-1-propane-sulfonic acid)/0.05 M ammonium hydroxide were added to each sub-sample. Analyses were performed using competitive ligand equilibration–adsorptive stripping voltammetry (CLE-AdSV) technique, by using 884 Professional VA Metrohm (Herisau, Switzerland) instrument, according to the following operating conditions: N_2_ purge time: 300 s; adsorption potential: − 0.1 V; deposition time: 60 s; equilibration time: 8 s; potential step time: from − 0.3 to − 0.75 V; scan mode: sampled DC; frequency: 10 Hz; voltage step: 4 mV; stirrer speed: 2000 min^−1^.

### Organic ligands identification by HPLC–ESI–MS/MS

Sample preparation followed the extraction and the preconcentration procedures by solid-phase extraction (SPE) technique developed by our group in a previous study (Rivaro et al. 2021). In particular, 50 mL of sample were loaded onto C18 SPE cartridges 500 mg, 3 mL (Supelclean™ ENVI™—18, Supelco®). Conditioning step consisted in 3 mL of methanol (MeOH, HPLC grade, VWR, Radnor, PA, USA) and 3 mL of Milli-Q water (Millipore, El Paso, TX, USA) which was acidified at pH ~ 2 with HCl (Merck). The solid phase was washed by loading 10 mL of acidified Milli-Q water and then dried using a N_2_ flow for 30 min. The elution was carried out with 3 mL of MeOH and the eluate dried by N_2_ flow, then stored at − 20 °C until the analysis. Before the analysis, 50 μL of a 0.1% (*v*/*v*) formic acid (Carlo Erba Reagents, Milan, Italy) solution in water were added to the dry sample. Afterward, 10 μL of sample were taken and diluted 1:1 with the same solution used before, then centrifuged for 5 min at 13,000 rpm. The structural information of the organic ligands was carried out by a micro high -performance liquid chromatography–electrospray ionization–tandem mass spectrometry (HPLC–ESI–MS/MS) using a HPLC 1100 series from Agilent Technologies (Santa Clara, California, USA) equipped with an autosampler and an Agilent Technologies XCT trap LC/MSD mass spectrometer, equipped with a high capacity ion trap. Full details of the procedure and of the instrumentation are given in Rivaro et al. (2021). Ferrioxamine E (FOE) (Merck, Darmstadt, Germany) and deferoxamine mesylate (DFMO) (Merck, Darmstadt, Germany) were used as standards for evaluating the presence of siderophore-type ligands in our samples based on their MS/MS fragmentation pattern. FOE and DFMO are two of the few commercial siderophore standards available.

### Dissolved oxygen, total alkalinity, pH, and nutrient analysis

Winkler method with a potentiometric detection of the end point of the titration was used to determine O_2_ on board (Grasshoff et al. 1983). Automated titroprocessor (Methohm 719, Herisau, Switzerland) was employed.

Total alkalinity (*A*_*T*_) and pH measurements were carried out using the methods described in Rivaro et al. (2010). Routine analyses of certified reference materials (batch 191, provided by A. G. Dickson, Scripps Institution of Oceanography) ensured the precision (± 3 μmol kg^−1^) and the accuracy (± 4 µmol kg^−1^) of the measurements. Potentiometric pH measures employed a combination glass/reference electrode with an NTC temperature sensor. The pH was expressed on the pH total scale (i.e., [H^+^] as moles per kilogram of seawater, pH_T_). The tris(hydroxymethyl)aminomethane (TRIS)/HCl buffer (batch 28, provided by A. G. Dickson, Scripps Institution of Oceanography) was used to standardize the electrode. The precision of the pH measurement was ± 0.007 units and was evaluated by repeated analysis of the *A*_*T*_ certified material.

Nutrients were determined using a five-channel continuous flow Technicon® Autoanalyzer II. The accuracy and the precision of the method were checked by certified reference material (CRM) MOOS-3 (seawater certified reference material for nutrients) (Clancy et al. 2014). The precision of the method was estimated by analyzing five homogeneous aliquots of the CRM, and it was ± 0.10 μM for NO_3_^−^, ± 0.01 μM for NO_2_^−^, ± 0.10 μM for NH_4_^+^, ± 0.30 μM for Si(OH)_4_ and ± 0.07 μM for PO_4_^3−^. The measured nutrients in the CRM MOOS-3 were not significantly different (*p* < 0.05) from the certified values.

### Data processing

The Fe speciation results were obtained following the calculations and the processing proposed by Gerringa et al. (2014).

The pH and *A*_*T*_ values measured at 25 °C have been recalculated at in situ conditions using the CO_2_SYS program (Pierrot et al. 2006). Equilibrium constants of CO_2_ (*K*_1_ and *K*_2_) of Millero et al. (2006) and pH_T_ scale (Dickson et al. 2007) together with CTD data (temperature, salinity, and pressure) were used for the calculations. In situ *C*_*T*_ and pCO_2_ values have been calculated as well.

Principal component analysis (PCA) was applied to the dataset in order to explore the correlations between the dFe, the Fe speciation data and the measured environmental parameters (temperature, salinity, fluorescence, O_2_, *A*_*T*_, *C*_*T*_, pH, pCO_2_, nutrients, Chl-*a*, and phaeopigments) in samples. The data of Chl-*a* and phaeopigments (Phaeo) together with a full description of the physical structure of the water column were already published in Bolinesi et al. (2020) and Rivaro et al. (2020). Data were normalized by log-transformation; then, the data matrix was processed after autoscaling the data using the R based software CAT (Leardi et al. 2017).

## Results

### Environmental conditions and biogeochemical properties

**Fig. 2:**
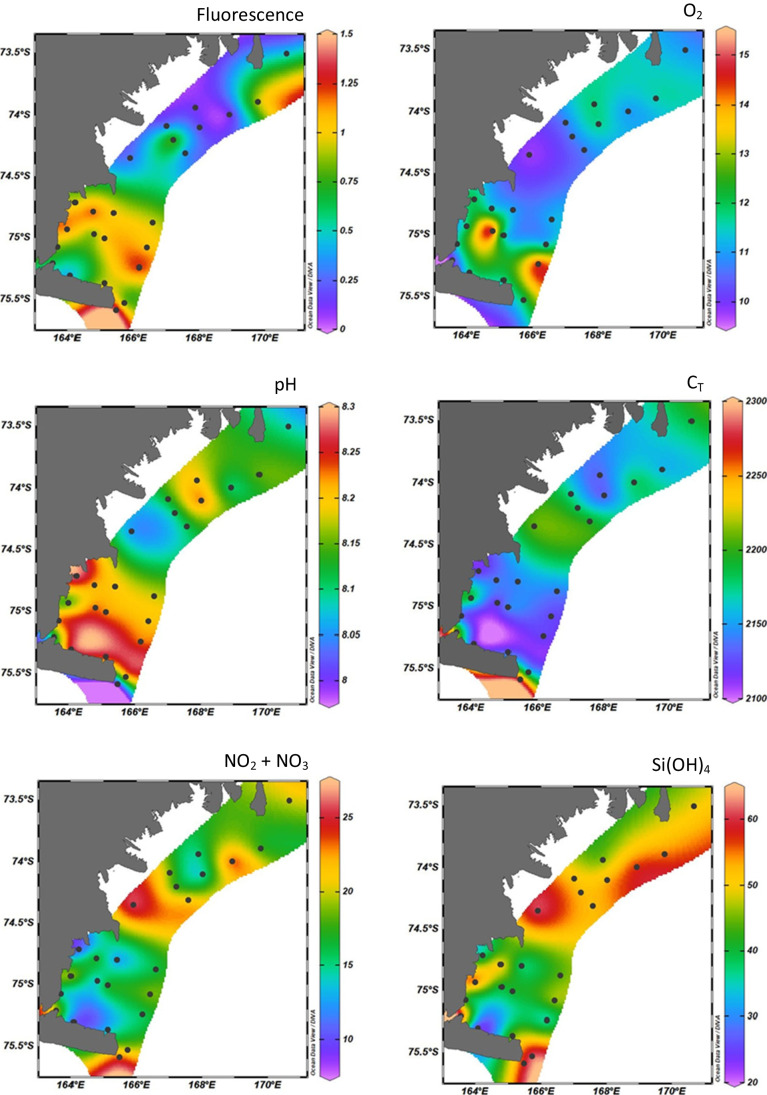
Horizontal distribution of **A** fluorescence (μg L^−1^); **B** dissolved oxygen (O_2_, mg L^−1^); **C** pH; **D** total inorganic carbon (*C*_*T*_, µmol kg sw.^−1^); **E** nitrate (NO_2_ + NO_3_, µM); **F** silicate (Si(OH)_4_, µM)

All data are reported in Table 1. Boxplots of temperature, salinity, fluorescence, O_2_, *A*_*T*_, pH, nutrients, and dFe are displayed in Fig. 2.

**Table 1:** Biogeochemical data

Area	Station	Depth (m)	O_2_ (mg L^−1^)	Temperature (°C)	Fluorescence (μg L^−1^)	Salinity	PO_4_ (μM)	Si(OH)_4_ (μM)	NO_2_ + NO_3_ (μM)	*A*_*T*_ (μmol kg sw − ^1^)	pH	*C*_*T*_ (μmol kg sw ^−1^)	pCO_2_ (µatm)
TNB	2	30	/	− 1.66	1.489	34.52	1.96 ± 0.11	57.2 ± 2.5	23.8 ± 2.3	2376 ± 4	7.98	2283	469
TNB	3	20	11.9	− 0.48	0.813	34.38	1.63 ± 0.18	62.3 ± 1.1	19.9 ± 0.7	2348 ± 4	8.21	2162	259
TNB	6	20	9.80	− 1.40	0.641	34.53	1.69 ± 0.09	63.1 ± 3.6	20.9 ± 1.5	2371 ± 3	8.09	2236	354
TNB	7	20	11.7	− 0.27	0.412	34.36	0.76 ± 0.03	23.3 ± 3.4	10.5 ± 1.07	2326 ± 2	8.26	2118	222
TNB	9	10	11.7	0.73	0.926	34.20	0.78 ± 0.02	45.3 ± 0.1	13.2 ± 0.1	2339 ± 4	8.25	2130	234
TNB	12	35	11.6	− 0.33	1.166	34.39	1.47 ± 0.06	56.6 ± 0.4	19.8 ± 0.2	2352 ± 4	8.14	2197	314
TNB	14	20	12.2	1.74	1.476	34.28	0.71 ± 0.05	41.7 ± 0.4	9.9 ± 0.2	2355 ± 3	8.29	2119	214
TNB	15	40	12.1	1.63	0.936	34.37	0.74 ± 0.01	36.1 ± 0.8	9.9 ± 0.2	2356 ± 3	8.29	2116	209
TNB	16	20	11.3	0.43	1.211	34.35	1.20 ± 0.03	51.7 ± 1.3	17.2 ± 0.4	2333 ± 0	8.21	2142	256
TNB	17	20	11.8	− 0.04	0.828	33.88	0.89 ± 0.04	39.2 ± 0.4	13.1 ± 0.3	2309 ± 2	8.24	2115	239
TNB	19	20	11.3	0.28	1.061	34.38	1.03 ± 0.01	41.5 ± 0.3	17.3 ± 1.7	2342 ± 4	8.22	2147	251
TNB	20	20	11.1	0.64	1.005	34.20	0.89 ± 0.03	37.5 ± 0.4	12.5 ± 1.4	2331 ± 2	8.20	2144	263
TNB	21	30	11.2	− 0.14	0.951	34.10	1.05 ± 0.01	44.1 ± 0.2	16.1 ± 0.8	2313 ± 4	8.18	2144	277
TNB	22	15	11.2	0.34	1.068	34.04	0.83 ± 0.04	46.8 ± 0.9	18.8 ± 0.5	2310 ± 2	8.21	2125	259
TNB	23	15	11.2	0.56	1.264	33.98	0.78 ± 0.01	35.2 ± 0.4	15.7 ± 0.5	2320 ± 1	8.23	2124	247
AMG	27	34	10.7	− 0.62	0.260	34.26	1.48 ± 0.01	51.9 ± 0.5	19.5 ± 0.3	2323 ± 3	8.13	2175	317
AMG	28	30	11.8	− 0.57	0.139	34.12	1.22 ± 0.01	45.1 ± 0.9	14.9 ± 0.1	2312 ± 2	8.20	2134	260
AMG	30	35	11.0	− 1.05	0.450	34.24	1.56 ± 0.01	49.4 ± 0.6	20.1 ± 0.5	2318 ± 2	8.09	2188	351
AMG	31	25	11.6	− 1.00	0.958	34.21	1.34 ± 0.13	54.8 ± 1.9	17.2 ± 0.1	2312 ± 7	8.16	2156	293
AMG	32	28	10.9	− 1.31	0.061	34.36	1.65 ± 0.04	57.8 ± 0.9	24.4 ± 1.8	2318 ± 5	8.09	2186	343
AMG	33	28	11.8	− 0.18	0.194	34.06	1.15 ± 0.01	53.1 ± 0.3	13.8 ± 1.6	2324 ± 0	8.24	2129	239
AMG	34	29	10.7	− 0.54	0.848	34.23	1.35 ± 0.01	52.7 ± 0.4	17.4 ± 0.1	2319 ± 14	8.10	2181	341
AMG	35	27	10.7	− 0.81	0.351	34.21	1.49 ± 0.01	52.4 ± 0.9	21.7 ± 0.4	2338 ± 7	8.12	2198	336
AMG	37	31	9.80	− 0.86	0.277	34.37	1.80 ± 0.02	61.2 ± 0.1	25.8 ± 0.1	2331 ± 6	8.06	2209	380

Temperature varied from − 1.66 to 1.74 °C at TNB and from − 1.31 to 1.03 °C at AMG; salinity from 33.88 to 34.53 and from 34.06 to 34.47 at TNB and at AMG, respectively. The fluorescence provided an indication of the abundance of Chl-*a*, i.e., the abundance of phytoplankton, and ranged from 0.412 to 1.489 and from 0.061 to 0.958 μg L^−1^, respectively.

O_2_ ranged from 9.8 to 12.2 and from 9.8 to 11.9 mg L^−1^ at TNB and AMG, respectively. Almost all stations sampled were near or above the O_2_ saturation level (97–111%), except station 6 and 37 where the saturation was 83% and 87%, respectively. Total alkalinity (*A*_*T*_) varied from 2309 to 2375 μmol kg sw^−1^ and from 2312 to 2350 μmol kg sw^−1^, and pH from 7.98 to 8.29 and from 8.06 to 8.25. All pCO_2_ values of the subsurface waters were below pCO_2_ atmospheric level (401.0 ppm https://www.exploratorium.edu/sites/default/files/files/SouthPoleCO2data_2020.pdf), varying between 210 and 381 µatm, with the lowest values calculated at stations 14 and 15. Total inorganic carbon (*C*_*T*_) varied from 2115 to 2283 μmol kg sw^−1^ and from 2128 to 2209 μmol kg sw^−1^ at TNB and AMG, respectively. TNB was characterized by a wider range of temperature with positive values too and by a wider range of salinity than AMG (Bolinesi et al. 2020). These observations are consistent with fluorescence, pH, and O_2_ data. Maximum of pH and *C*_*T*_ and pCO_2_ minima were recorded in those stations where both high O_2_ and fluorescence values were found.

Nutrients were never fully depleted in both investigated areas; the lowest concentration of NO_3_^−^ and PO_4_^3−^ were recorded at station 14 at TNB (9.9 and 0.71 µM, respectively). Nitrate ranged from 9.90 to 23.8 μmol kg^−1^ (TNB) and from 10.4 to 25.8 μmol kg^−1^ (AMG), PO_4_^3−^ from 0.71 to 1.96 μmol kg^−1^ (TNB) and from 0.84 to 1.80 (AMG) and silicate from 23.3 to 63.1 μmol kg^−1^ (TNB) and from 36.1 to 61.2 μmol kg^−1^ (AMG).

### Total dissolved iron and organic speciation analysis

Total dissolved iron and iron speciation data are reported in Table 2. Dissolved iron concentrations ranged from 0.4 to 2.5 nM at TNB area and from 0.5 to 2.0 nM at AMG area, respectively. The range is greater than the data reported for the open Southern Ocean (Boyd and Ellwood 2010; Ellwood et al. 2020), but comparable with the data collected in TNB during summer season (Grotti et al. 2001; Rivaro et al. 2012, 2019). All the samples showed about > 99% of the dFe bound to organic ligands (*L*), in accordance with other Fe speciation studies conducted in the Southern Ocean (Ibisanmi et al. 2011; Rivaro et al. 2019). Organic ligands ranged from 1.1 to 6.9 nM for the TNB area and from 1.3 to 7.1 nM for the AMG area. No marked differences between the two areas were observed and the range was comparable with previous speciation studies conducted in TNB polynya (Rivaro et al. 2019). The concentration of *L* was always higher than the concentration of dFe. The difference between the concentration of *L* and dFe defines free ligands (*L*′), which represents the concentration of ligands with sites available to complex Fe. A small value of *L*′ suggests an almost total saturation of the available sites. The concentration of *L*′ displayed a wide range of values, from 0.3 to 6.2 nM at TNB and from 0.2 to 6.3 nM at AMG. Similarly to the other parameters, the two sampling sites showed no substantial differences nor a common coast-open sea trend. The *L*/dFe ratio also provides information on the saturation state of organic ligands: a value close to one corresponds to ligands relatively saturated with Fe and indicates a low capacity of the ligands to complex and buffer further Fe additions (Thuróczy et al. 2010, 2011). On the contrary, a relatively high value (> 5) suggests that the ligand pool is undersaturated, and it can therefore buffer further Fe additions, increasing the potential solubility of Fe by keeping it in the dissolved phase (Thuróczy et al. 2012). The *L*/dFe ratio ranged from 1.1 and 9.3 for TNB area and from 1.1 and 8.8 for AMG area, suggesting highly variable conditions among the stations even within the same study area. The values obtained are in accordance with previous works both for the Terra Nova Bay area and for other regions of the Southern Ocean (Boye et al. 2001; Lannuzel et al. 2015; Rivaro et al. 2019; Gerringa et al. 2019). The log*K*’_Fe’*L*_ values (13.0–15.0) are similar for the two areas under examination, highlighting that the Fe is stably complexed with the natural organic ligands present in sea waters.

**Table 2:** Total dissolved iron concentration and iron speciation data

Station	Area	dFe (nM)	*L* (nM)	logK’_Fe’L_	SD_d_	SD_up_	*L*′ (nM)	*L*/dFe	Fe' (pM)	FeL (%)	logα_Fe’*L*_
2	TNB	2.5	2.8 ± 0.1	15.0 ± 0.5	NA	0.38	0.3	1.1	0.01	99.9	16.4
3	TNB	0.8	5.4 ± 0.1	13.3 ± 0.1	0.13	0.10	4.6	6.9	0.01	99.9	15.1
6	TNB	0.8	1.1 ± 0.1	14.7 ± 0.4	NA	0.40	0.3	1.4	0.01	99.9	15.8
7	TNB	0.6	5.3 ± 0.1	13.3 ± 0.1	0.06	0.06	4.7	9.1	0.01	99.9	15.0
9	TNB	0.8	2.2 ± 0.1	13.5 ± 0.4	0.64	0.26	1.3	2.6	0.02	99.9	14.8
12	TNB	1.2	1.6 ± 0.1	14.5 ± 0.5	0.74	0.26	0.4	1.4	0.01	99.9	15.7
14	TNB	0.9	1.9 ± 0.1	13.8 ± 0.1	0.23	0.15	1.0	2.2	0.02	99.9	15.0
15	TNB	1.4	1.7 ± 0.1	15.0 ± 0.4	NA	0.65	0.3	1.2	0.01	99.9	16.3
16	TNB	0.9	3.9 ± 0.1	13.5 ± 0.1	0.14	0.10	3.0	4.3	0.01	99.9	15.1
17	TNB	1.3	2.4 ± 0.1	13.8 ± 0.1	0.17	0.12	1.1	1.8	< LOD	99.9	15.2
19	TNB	1.0	2.6 ± 0.1	13.6 ± 0.5	0.71	0.26	1.5	2.5	0.01	99.9	15.0
20	TNB	1.5	3.9 ± 0.1	13.8 ± 0.1	0.12	0.09	2.4	2.7	0.01	99.9	15.4
21	TNB	1.7	3.6 ± 0.1	13.7 ± 0.1	0.20	0.14	1.9	2.1	0.02	99.9	15.3
22	TNB	0.8	6.9 ± 0.1	13.2 ± 0.1	0.10	0.08	6.2	9.3	0.01	99.9	15.0
23	TNB	0.4	2.8 ± 0.1	13.3 ± 0.0	0.64	0.24	2.4	7.1	0.01	99.8	14.7
27	AMG	1.1	1.6 ± 0.1	14.5 ± 0.1	0.88	0.27	0.5	1.4	0.01	99.9	15.7
28	AMG	0.8	7.1 ± 0.2	13.4 ± 0.1	0.05	0.05	6.3	8.8	0.01	99.9	15.3
30	AMG	0.5	2.3 ± 0.2	13.4 ± 0.2	0.57	0.25	1.8	4.7	0.01	99.9	14.8
31	AMG	2.0	2.4 ± 0.1	14.1 ± 0.4	0.20	0.14	0.4	1.2	0.04	99.9	15.4
32	AMG	0.9	3.2 ± 0.1	13.5 ± 0.1	0.60	0.34	2.2	3.4	0.04	99.9	15.0
33	AMG	0.7	2.7 ± 0.2	13.0 ± 0.4	NA	0.41	2.0	3.7	0.01	99.8	14.5
34	AMG	0.6	2.2 ± 0.1	13.5 ± 0.2	0.34	0.18	1.7	4.0	< LOD	99.9	15.9
35	AMG	0.9	2.8 ± 0.1	13.4 ± 0.1	0.28	0.17	1.8	3.0	0.02	99.9	14.9
37	AMG	1.1	1.3 ± 0.1	13.4 ± 0.5	NA	0.57	0.2	1.1	0.01	99.9	16.1

**Fig. 3:**
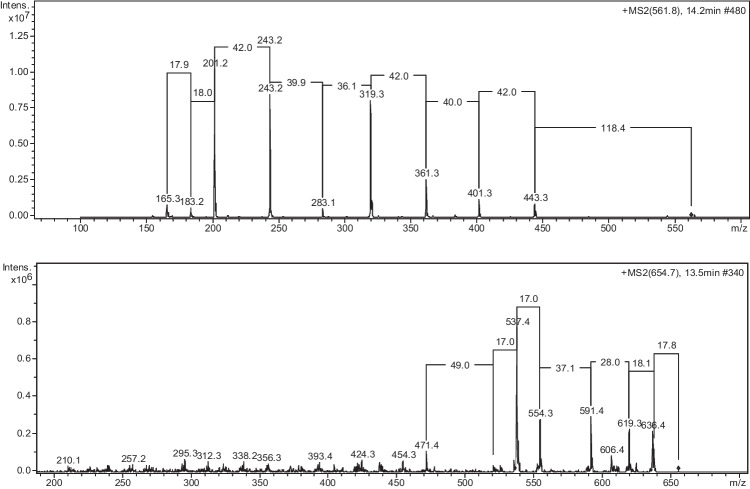
MS/MS spectra of deferoxamine mesylate (DFMO) and ferrioxamine E (FOE)

Concerning the HPLC–ESI–MS/MS results, the MS/MS spectra for DFMO and FOE standard are shown in Fig. 3.

**Fig. 4:**
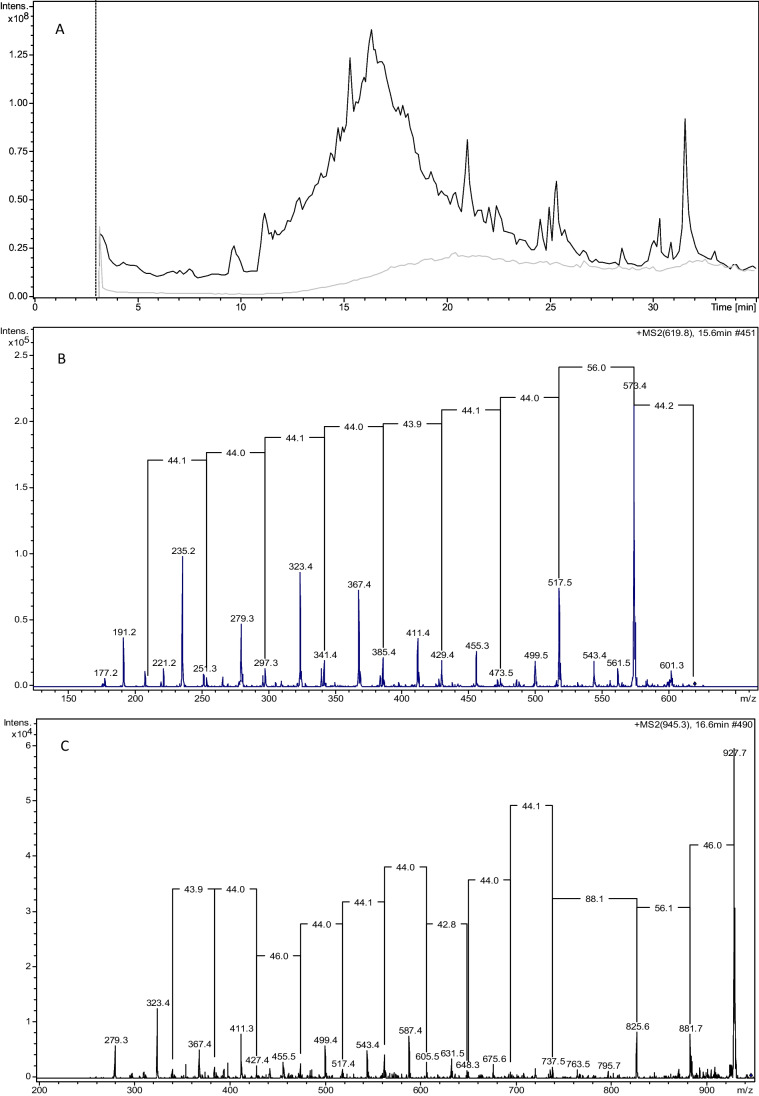
**A** Chromatogram of a seawater sample (black) and of the procedural blank (grey); **B** MS/MS spectra of some extracted ions

With regard to the samples, some peaks present only in the samples were identified by comparing the chromatograms of the samples with the procedural blank (Fig. 4A). In the MS/MS, no losses of the mass characteristic for DFMO and FOE standard were observed. On the contrary, the frequent loss of fragments with mass 19, 44, 46, 56, 64 was observed (Fig. 4B).

### Relationships between environmental features and dissolved iron speciation

**Fig. 5:**
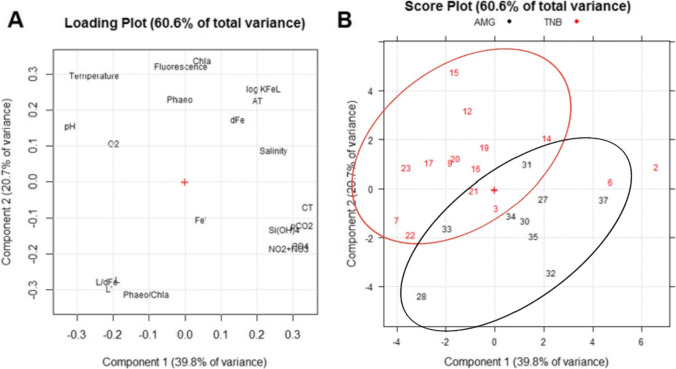
**A** Loading plot and **B** score plot obtained from the PCA analysis of the environmental analytical dataset of the TNB and AMG areas. The following abridgements were used for the variables: dissolved oxygen (O_2_), phosphate (PO_4_.^3−^), nitrate (NO_2_ + NO_3_), silicate (Si(OH)_4_), total inorganic carbon (*C*_*T*_), total alkalinity (*A*_*T*_), chlorophyll-*a* (Chl-*a*), phaeopigments (Phaeo), dissolved iron (dFe), free dFe (Fe’), organic ligands (*L*), and free organic ligands (*L*’)

Differences and similarities among the stations sampled in both areas were outlined through PCA. Two principal components were identified: PC1 explained 39.8% of the total variance, while PC2 explained a further 20.7%. Temperature, pH, and O_2_ loaded on the negative values of PC1 and positive values of PC2, while nutrients, *C*_*T*_ and pCO_2_ loaded on the positive values of PC1 and negative values of PC2 and, in particular, these variables were negatively correlated. On the contrary, fluorescence, Chl-*a* and Phaeo, salinity, *A*_*T*_ together with speciation data mainly loaded on the PC2. The loadings of the variables showed that *L*, *L*′, and *L*/dFe were negatively correlated with the other speciation parameters (dFe and log*K*’_Fe’*L*_) (Fig. 5A). As shown in the score plot (Fig. 5B), AMG and TNB stations essentially form two groups. In particular, the AMG stations are mainly distributed in the positive part of PC1 and those of TNB in the negative part and these also have a greater distribution along PC2. Moving along PC2, it is possible to observe that *L* concentration decreases from station 22 to station 15 and the log*K*’_Fe’L_ decreases from station 15 to station 22. The score plot highlights that some samples (stations 2 and 6) did not fall into the clusters of samples collected at TNB. Station 2 was characterized by the highest dFe, fluorescence, PO_4_^3−^, *A*_*T*_, *C*_*T*_, and pCO_2_ and by the lowest O_2_, temperature and pH. Station 6 had values intermediate between those of station 2 and those of the TNB cluster.

## Discussion

The evaluation of the dependence of Fe speciation on physical and biological variables is one of the objectives of the CELEBeR project. The AMG area extends from the Aviator Ice tongue to the Mariner Ice tongue near Coulman Island. TNB is bounded by three steep glacier valleys, the Reeves Glacier and Priestley Glacier draining into the Nansen Ice Sheet (NIS) and the David Glacier terminating in the Drygalski Ice Tongue (DIT) (Rivaro et al. 2020). The TNB area has been extensively studied for years by the international scientific community due to its relevance in terms of primary production during the summer time (Saggiomo et al. 1998; Tremblay and Smith 2007; Smith et al. 2010; Mangoni et al. 2017). Phytoplankton blooms develop later in the year than in the Ross sea polynya, and they are dominated by diatoms (Saggiomo et al. 2017). Instead, the AMG area, despite being close to TNB, was investigated for the first time in a systematic manner during the CELEBeR project and therefore the data discussed in this work constitute the first available dataset.

### Relationship between iron and coastal biogeochemistry in the Ross Sea

Physical and biological characteristics of the surface waters and the circulation patterns of both areas during the sampling have been already reported and discussed in Bolinesi et al. (2020), Rivaro et al. (2020), and Zaccone et al. (2020). TNB and AMG presented different physical and biogeochemical properties although neighboring coastal systems. The PCA highlighted a transition (Fig. 5A and B) leading to the separation of samples collected at TNB (that have low nutrients concentration, *C*_*T*_ and high pH, temperature, and O_2_ samples) from those collected at AMG. The distribution of the samples of TNB along PC2, on which fluorescence, Chl-*a*, Phaeo, and Phaeo/Chl-*a* ratio mainly weigh, highlights the higher contribution of biological activity in this area in defining the chemical properties. TNB was first sampled, and the sampling time was very short (13 days). As a consequence, the PCA results do not reflect the typical evolution of biogeochemical parameters accompanying the seasonal phytoplankton bloom from earlier in season (AMG) to later in season (TNB).

The range of dFe was lower than the particulate iron (pFe) measured in the same samples (from 0.41 to 8.70 nM) and, similarly to pFe, a coast-open sea trend was not found, confirming the spatial heterogeneity (Rivaro et al. 2020). The higher dFe concentration found in the subsurface waters compared to offshore waters can reflect a different input either from land or from ice melting. The combination of salinity with δ^18^O allowed us to establish that sea ice melting was relevant in many stations except for stations 2, 6, 7, 15, 16, 17, and 19 in TNB (Rivaro et al. 2020). On the contrary, it was not relevant for most of the stations in AMG area. These observations supported the hypothesis drawn by Bolinesi et al. (2020) on the role of the thickness and stability of the upper layer of the water column in determining the observed different distribution of phytoplankton in the two areas. The low correlation (Spearman correlation) between dFe and *S* and dFe and δ^18^O (*p* = 0.762, *r* =  − 0.069 and *p* = 0.084, *r* =  − 0.360, respectively) seemed to suggest different sources of dFe for the surface waters. The high dFe concentration measured in stations 20 and 21 can have been released from sea- ice melting, whereas in station 2, characterized by a high salinity value, other sources such as atmospheric fall out or remineralization from organic matter in the upper mixed layer could have added Fe. TNB is defined as a coastal polynya, i.e., an area of sea that remains substantially ice-free throughout the year, due to the action of katabatic winds. Wind transport could therefore represent an additional source of iron for surface waters with.

Taxonomic variability in nitrogen (N), phosphorus (P), and silica (Si) drawdown ratios can have important biogeochemical implications. Si:N and N:P ratios were calculated plotting the NO_3_ + NO_2_ + NH_4_ concentration versus the Si(OH)_4_ or PO_4_^3−^ concentration. The slope of the Si:N disappearance ratio resulted in 2.1 and 1.1 for TNB and AMG respectively. The slope of the N:P disappearance ratio resulted in 9.1 and 17.6 for TNB and AMG respectively. The TNB ratio is consistent with the values reported for diatoms dominated waters, whereas the AMG value suggests lower diatoms and higher haptophytes contributions to phytoplankton biomass. PCA score plot shows that the biogeochemical features of stations 28 and 33 are more similar to samples from TNB than those from AMG. This is confirmed by the N:P ratio that resulted significantly lower (11.2) than that calculated for AMG and closer to the TNB ratio.

The algal assemblage composition together with the physiological strategy of the micronutrient uptake condition the Si:N ratio value. In fact, *Phaeocystis* sp. does not assimilate silicic acid, whereas diatoms require silicic acid for the production of biogenic silica frustules. Moreover, the Si:N uptake ratio is about 1 under Fe-replete conditions, but it increases to values above 2 under Fe-deplete conditions, because the nitrate uptake is reduced (Hutchins and Bruland 1998). Thus, the observed Si:N ratio suggests that TNB was near to Fe-depletion during our sampling despite the high dFe concentration. On the contrary the AMG area seems Fe-replete. This hypothesis can be supported by the results of Bolinesi et al. (2020) who found a slightly higher Phaeo/Chl-a ratio in TNB than in AMG (Bolinesi et al. 2020).

### Implication of the iron speciation for iron bioavailability

One of the problems in the study of Fe speciation in natural waters is the identification of organic ligands. Several compounds have been included such as siderophores, pigment-like compounds including the heme group, humic substances, and polysaccharides. However, their relative importance in the Fe speciation, biogeochemistry, and bioavailability has not been completely defined (Laglera et al. 2020). Electrochemistry is one of the method most often employed for metal speciation studies in seawater. The CLE-AdSV has been used to estimate the Fe-binding capacity of ligands. The concentration of ligands and the conditional stability constant of their Fe complexes depend on the chosen artificial ligand and on the composition of the sample matrix. Thus, we must consider that the data obtained with different artificial ligands could be different accordingly to the used artificial ligands. Moreover, the CLE-AdSV gives information on the Fe-binding capacity of seawater at pH, temperature, and dFe concentration of the sample (Gerringa et al. 2021). Ligands concentration was always higher than the dFe concentration, and it displayed a coastal-offshore increasing gradient at TNB. The *L*/dFe ratio had values between 1 and 5 in almost the majority of the samples, outlining an intermediate condition between saturation and undersaturation of the ligands. Nevertheless, stations 2, 15, 31, and 37 had *L*/dFe values of about 1 and low values of *L*′, implying that in these samples the Fe-binding sites are unavailable for Fe complexation. On the contrary, stations 3, 7, 22, 23, and 28 displayed *L*/dFe above 5 and the highest *L*′ values suggesting that all Fe-binding sites were unsaturated. The high *L*/dFe values are the consequences of the highest *L* concentration and of the lowest dFe concentration. In any case, the stability of the dFeL complexes is outlined by the high logK’_Fe’*L*_ values (13.8 ± 0.6).

Generally, log*K*’_Fe’*L*_ values (13.0–15.0) were higher than those obtained from the analysis of samples collected in the same area in previous surveys (12.1–13.6) (Rivaro et al. 2019). The highest log*K*’_Fe’*L*_ were calculated for the coastward stations of TNB (stations 2, 6, and 15), suggesting the presence of particularly stable complexes between Fe and natural ligands in sea water. According to the Fe-binding affinity, the ligands can be divided into different classes: *L*_1_ includes stronger ligands, with log*K*’_Fe’*L*_ = 12–13 or higher, while *L*_2_ to *L*_4_ types gather weaker ligands, with log*K*’_Fe’*L*_ = 10–12 or lower (Vraspir and Butler 2009; Ibisanmi et al. 2011). From the values obtained in this study, it can be stated that the ligands present in our samples belong to class *L*_1_.

**Fig. 6:**
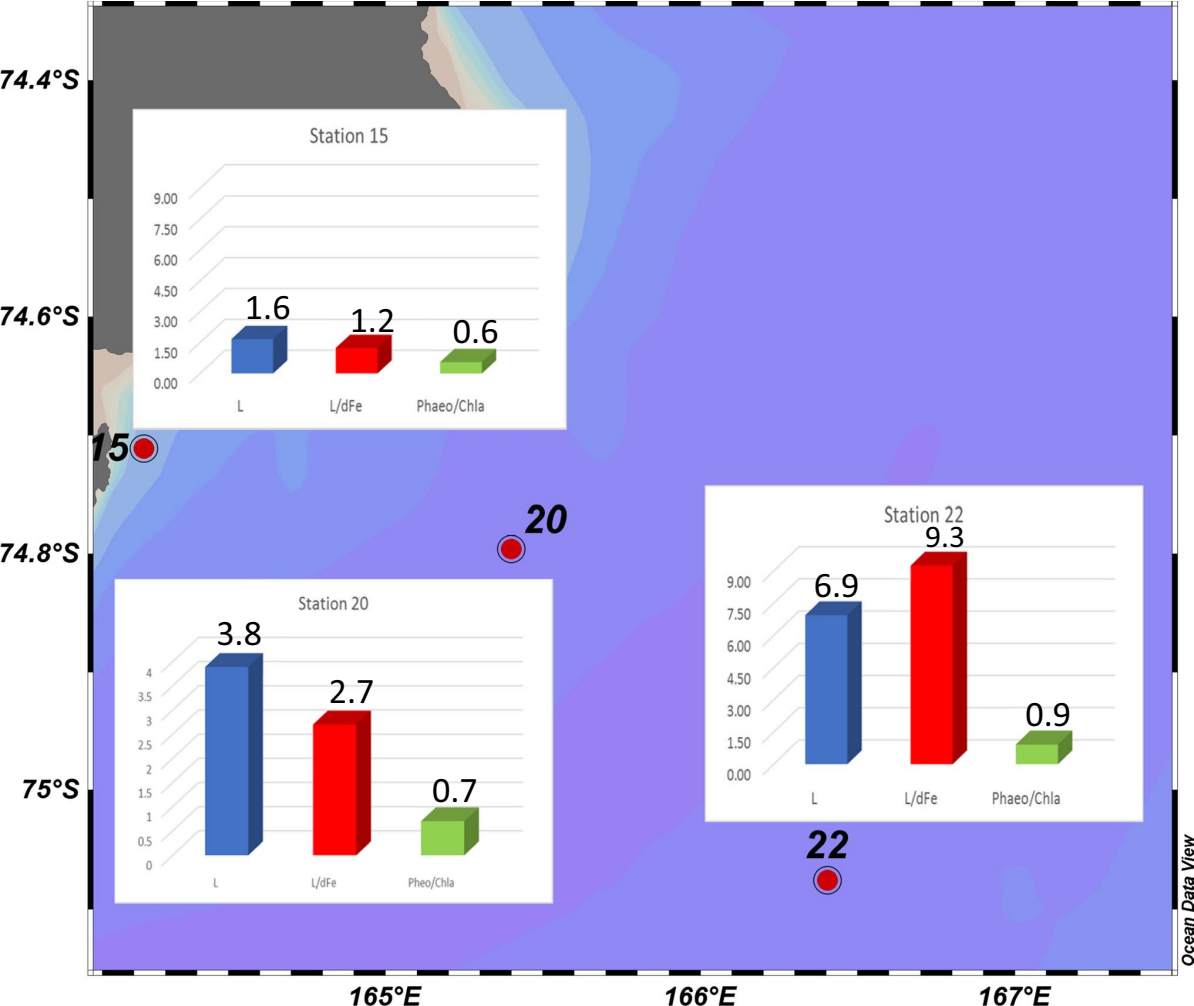
Histograms relating to ligand concentration (*L*, nM), *L*/dFe ratio, and phaeo/Chl-*a* ratio of stations 15, 20, and 22 sampled at TNB

Similarly to what found by Gerringa et al. (2019) in the Ross sea polynya and the eastern Ross sea shelf area, no correlation between both *L* and Chl-*a* and *L* and fluorescence was evidenced. On the contrary, the positive and significant correlation between *L* and Phaeo/Chl-*a* ratio (*r* = 0.512; *p* = 0.013) suggested that dFe-binding organic ligands could be released during grazing. In fact, an increase of Phaeo/Chl-*a* ratio suggests an advanced bloom phase and/or and increasing of grazing, since phaeopigments are a decomposition product of Chl-*a*. This observation is depicted in Fig. 6, where ligand concentrations, *L*/dFe ratio and Pheo/Chl-*a* ratio are compared at three stations (15, 20, and 22) in the TNB area selected based on PCA results.

Grazing has recently shown to be important for the recycling of dFe in Antarctic waters. Laglera et al. (2020) in the course of the Fe fertilization experiment hypothesized that during the grazing stage, sloppy feeding while copepod grazing of cells and pellets was the major process of release of dFe and ligands mostly in the form of strong FeL_1_ complexes (Laglera et al. 2020). Moreover, high phytoplankton activity may influence the organic matter (OM) availability and, in turn, the prokaryotic activity, which can release Fe-binding ligands (Hassler et al. 2011). Grazing processes can thus not only remineralize biogenic Fe, but also alter the chemical speciation of Fe in marine waters, greatly affecting phytoplankton species composition during phytoplankton bloom particularly in Fe-limited waters (Sato et al. 2007). Similarly to the Hassler et al. (2017) study, in a previous survey carried out in TNB, we have found that ligand distribution did not co-vary with Chl-*a* concentration, but it negatively and significantly co-varied with prokaryotic biomass, suggesting a role of microbial activities in determining *L* distribution (Rivaro et al. 2019).

The metabolic activity of prokaryotes involved in the biogeochemical cycles was investigated in the framework of CELEBeR project activities (Zaccone et al. 2020). Dissolved organic matter (DOM) was not included in the sampling strategy, and we have only few data that refers to dissolved organic carbon. On the contrary, key microbiological parameters (the proteasic, glucosidasic, and phosphatasic activities; the prokaryotic abundance; and biomass) were evaluated in relation to quantitative and qualitative characteristic of particulate organic matter. High variability of the microbial parameters was observed with the highest prokaryotic biomass in the coastward stations (Zaccone et al. 2020).

Many authors have hypothesized that the strongest ligands often found in natural seawater are siderophores exudated by prokaryotes (Vraspir and Butler 2009, Gledhill and Buck 2012; Laglera et al. 2020). This hypothesis is consistent with the high prokaryotic biomass and with the dominance of diatoms in phytoplankton biomass found during the CELEBeR sampling. In fact, diatoms exploit particular and complex metabolic strategies to uptake Fe complexed to strong ligands (Gao et al. 2021). The uptake involves endocytosis of the siderophore type of complex within the cell, after reducing the complexed Fe, next to the chloroplast (Kazamia et al. 2018). In particular, Fe starvation–induced protein 1 (ISIP1) was identified through reverse genetic, and considered necessary for the endocytosis and assimilation of the siderophore (Coale et al. 2019).

An effort has been done in order to deeply investigate the presence of siderophore-like ligands by HPLC–ESI–MS/MS analyses. The analyses were carried out on the samples collected in TNB due to the higher biological effect on the Fe speciation parameters highlighted by PCA results than in AMG. We used DFMO and FOE as siderophore standards during the development of our method on the basis of the study by Mawji et al. (2008) who analyzed by HPLC–MS/MS subsurface samples of Atlantic waters and found hydroxamate-type siderophores. Nevertheless, we did not observe the loss of the fragments characteristic of DFMO and FOE in our MS/MS spectra. Therefore, we can exclude the presence of these specific siderophore structures in our samples. However, we observed a frequent loss of fragments having mass 19, 44, 46, 56, 64 similarly to that found by Zajdowicz et al. (2012) for an heterogeneous class of siderophores containing two citric acid subunits, with the central α-hydroxycarboxylic acid moiety of each citrate serving as an iron-complexing ligand (Budzikiewicz 2005). We are not yet able to quantify the contribution of this class of substances to the pool of organic ligands, but at this stage we can only hypothesize their nature based on the comparison of conditional stability constants values and MS/MS results.

The chromatograms and MS/MS spectra did not show signals characteristic of extracellular polymeric substances (EPS). These are an important component of the DOM in the sea ice, playing several biological roles (Krembs et al. 2002). They are mainly composed by carbohydrates and they have affinity for Fe, influencing its biogeochemical cycle, speciation, and bioavailability (Gledhill and Buck 2012; Rivaro et al. 2021). Thus, EPS-rich meltwaters could enhance the concentration of bioavailable Fe in the surface waters supporting high levels of primary production. The results available in the literature obtained by CLE-AdCSV give different insights regarding the class of ligands to which EPSs belong, depending on the log*K*’_Fe’*L*_ values obtained. For example, Hassler et al. (2011) assign them to class *L*_2_–*L*_4_, whereas Norman et al. (2015) consider them borderline between class *L*_1_ and *L*_2_. Since the ligands in our samples belong to the *L*_1_ class, with log*K*’_Fe’*L*_ values greater than 13, we can assume that the contribution of EPS to the ligand pool is not relevant. Furthermore, the sampling took place in mid-January, when the pack was already melted, and the DOM released into the surface waters. We can assume that the EPS being mostly composed of polysaccharides were rapidly consumed by microorganisms as part of the labile fraction of the DOM (Biersmith and Benner 1998). These findings agree with the absence of correlation found between *L*, dFe, and sea ice meltwater and with the calculated log*K*’_Fe’*L*_.

## Conclusion

The distribution of dFe and its speciation in the subsurface waters sampled in coastal areas of the western Ross sea during austral summer 2017 was investigated. In particular, the study was done on the well-studied site of TNB polynya and on the neighboring area of AMG, which to date has not been studied in a systematic way. The chemometric approach to the analysis of the biogeochemical dataset outlined that TNB and AMG area were different in terms of chemical, physical, and biological parameters and confirmed the general higher role of the biological activity at TNB than at AMG, although close coastal systems.

The high dFe concentration found in both investigated areas reflected the Fe input either from land or from ice melting. The spatial heterogeneity and complexity in Fe distribution and speciation at a horizontal length scale of about 10 km found in a previous study has been confirmed. The Si:N ratio suggested that TNB was near to Fe depletion during our sampling despite the high-dFe concentration, whereas the AMG area seemed Fe replete.

The study of the organic speciation is a key factor in understanding the biogeochemical cycle of Fe in the shelf area of the western Ross sea, which is one most productive area of the Southern Ocean. CLE-AdSV results showed high *L* concentration and very high logK’_Fe’*L*_ values, which suggested a high stability of the Fe complexes. The positive and significant correlation between *L* and Phaeo/Chl-*a* ratio suggested that dFe-binding organic ligands could be released during grazing.

For the first time, a coupling between voltammetric and mass spectrometry data has been carried out in studying the Fe speciation in the western Ross sea. HPLC–ESI–MS/MS analyses helped us to better understand the nature of the highly stable *L* highlighting the presence of a heterogeneous class of siderophores in organic ligands pool. Unfortunately, due to the lack of siderophore standards, we could not quantify the contribution of this class of substances, but we could only hypothesize their nature based on the comparison of stability constants values and MS/MS results.

However, our data open a window to better understand the Fe biogeochemical cycle and speciation in the Antarctic seawater that could be useful to predict changes in Fe availability in the future. In fact, climate-driven changes in the productivity biomass of phytoplankton and microbial communities are virtually certain to impact Ross sea Fe biogeochemistry, by modifying the balance among biological uptake, chemical speciation, vertical export, and organic matter recycling.

## Supplementary Information

Below is the link to the electronic supplementary material.

Supplementary file1 (DOCX 13 KB)

## Data Availability

All data generated or analyzed during this study are included in this published article (and its supplementary information files).
