# Supplementary material for: Combining voltammetric and mass spectrometric data to evaluate iron organic speciation in subsurface coastal seawater samples of the Ross sea (Antarctica)
Source: Environ Sci Pollut Res Int. 2022 Nov 12;30(10):26718–34. doi: 10.1007/s11356-022-23975-w (PMC9995544; doi:10.1007/s11356-022-23975-w)
Supplement: Supplementary file 1 — Supplementary file1 (DOCX 13 KB) [file 11356_2022_23975_MOESM1_ESM.docx]

| **Station** | **Latitude [degrees south]** | **Longitude [degrees east]** | **Sampling Date** |
| --- | --- | --- | --- |
| 2 | -75.5858 | 165.4712 | 09-01-2017 |
| 3 | -75.5273 | 165.7213 | 09-01-2017 |
| 6 | -75.2055 | 163.5515 | 10-01-2017 |
| 7 | -75.2982 | 164.0772 | 10-01-2017 |
| 9 | -75.3630 | 165.1157 | 10-01-2017 |
| 12 | -75.0720 | 163.7043 | 13-01-2017 |
| 14 | -74.9277 | 163.9963 | 13-01-2017 |
| 15 | -74.7115 | 164.2308 | 13-01-2017 |
| 16 | -74.7837 | 164.7640 | 13-01-2017 |
| 17 | -74.9670 | 164.7920 | 14-01-2017 |
| 19 | -75.0050 | 165.1255 | 14-01-2017 |
| 20 | -74.7960 | 165.3995 | 14-01-2017 |
| 21 | -74.8748 | 166.5780 | 14-01-2017 |
| 22 | -75.0757 | 166.4043 | 15-01-2017 |
| 23 | -75.2368 | 166.1813 | 15-01-2017 |
| 26 | -74.1475 | 166.1753 | 20-01-2017 |
| 27 | -74.0868 | 167.0123 | 20-01-2017 |
| 28 | -73.9363 | 167.8852 | 20-01-2017 |
| 30 | -73.50074 | 170.65714 | 20-01-2017 |
| 31 | -73.8875 | 169.7823 | 21-01-2017 |
| 32 | -73.9925 | 168.9287 | 21-01-2017 |
| 33 | -74.0983 | 168.0253 | 21-01-2017 |
| 34 | -74.1983 | 167.2097 | 21-01-2017 |
| 35 | -74.3113 | 167.58 | 21-01-2017 |
| 37 | -74.3475 | 165.8942 | 21-01-2017 |

Table S1. Sampling stations.
